# Orai3 is a predictive marker of metastasis and survival in resectable lung adenocarcinoma

**DOI:** 10.18632/oncotarget.13149

**Published:** 2016-11-07

**Authors:** Nazim Benzerdjeb, Henri Sevestre, Ahmed Ahidouch, Halima Ouadid-Ahidouch

**Affiliations:** ^1^ Laboratory of Cellular and Molecular Physiology, LPCM: EA 4667, SFR CAP-SANTE (FED 4231), UFR of Sciences, Amiens, France; ^2^ Department of Pathology, Amiens University Hospital, Amiens, France; ^3^ Department of Biology, Ibn Zohr University, Agadir, Morocco

**Keywords:** orai3, lung adenocarcinoma, prognostic marker, survival

## Abstract

Orai3 channel has emerged as important player in malignant transformation. Indeed, its expression is increased in cancer and favors cell proliferation and survival by permitting calcium influx. In this study, Orai3 was overexpressed in lung adenocarcinoma as compared to their matched non-tumour samples and was associated with tumoural aggressiveness. Moreover, its expression was associated with estrogen receptor alpha (ERα) expression and visceral pleural invasion in multivariate analysis. Furthermore, both the overall survival (OS) median and the metastasis free survival (MFS) median of tumors with high Orai3 expression were lower than in low Orai3 expression regardless of cancer stage (35.01 months *vs.* 51.11 months for OS and 46.01 months *vs.* 62.04 months for MFS). In conclusion, Orai3 protein level constitutes an independent prognostic marker in lung adenocarcinoma, and a novel prognostic marker that could help selecting the patients with worst prognosis to be treated with adjuvant chemotherapy in resectable stage.

## INTRODUCTION

Lung cancer, particularly adenocarcinoma, is the leading cause of cancer deaths worldwide [1] and the most common histologic type of lung cancer [2]. Moreover, lung adenocarcinoma is known to be a very heterogeneous tumour, with over 90% of resected tumours [3]. For this reason, the accuracy of patient prognosis prediction of the international staging system is still insufficient with recurrence rate after radical surgery around 40% of early stage patients [4]. The use of chemotherapy [CT] after radical surgery is justified by increasing overall survival from 60 to 64% [5-7]. As the benefit of chemotherapy is moderate, it is important to select appropriate patients with resectable tumours. The chemotherapy is recommended for patients with stage II or III of lung adenocarcinoma, and also for patients with stage Ib with high risk of recurrence. Thus, it is necessary to implement new prognostic markers of high risk for recurrence that could help to identify the patients with worst prognosis [4]. Some histologic and immunohistochemical biomarkers have been proposed. In 2011, a lung adenocarcinoma classification based on predominant histologic pattern (lepidic, papillary, acinar, solid and micropapillary) has been proposed and is currently accepted [8]. The predominant histologic pattern: low (lepidic), intermediate (papillary, acinar) and high risk (solid, micropapillary), constitutes a predictive factor for recurrence in resectable stage lung adenocarcinoma [4]. Others immunohistochemical biomarkers, such as estrogen receptor alpha expression (ERα) expression, are associated with a poor prognosis in lung adenocarcinoma [9, 10]. Moreover, ERα also is an independent factor of recurrence in pT1a lung adenocarcinomas [11]. But few studies have discussed a biomarker involved in calcium metabolism.

Calcium (Ca^2+^) plays a crucial role in regulating several processes such as cell proliferation [12, 13] and apoptosis [14]. The scientific community, including our group, has shown an interest in Orai channels family because these latter are known to play a major pathway of calcium influx in epithelial cells. Three Orai isoforms have been characterized: Orai1, Orai2 and Orai3. Orai3 is a unique channel whose expression is restricted to mammals [15]. Several studies have reported the involvement of Orai3 in the complex machinery of carcinogenesis including breast, prostate and lung cancer [16-18]. Moreover, the expression of Orai3 is regulated by several factors. Indeed, in breast cancer, Orai3 is regulated by the ERα, thereby conferring apoptosis resistance and cell proliferation [16, 19, 20]. Indeed, silencing of ERα caused a significant decrease of Orai3 expression, calcium influx, and cell proliferation in vitro [19]. Furthermore, epidermal growth factor (EGF) stimulates Ca^2+^ influx into estrogen receptor-positive MCF-7 cells through Orai3 [16]. In lung cancer, we have previously reported an overexpression of Orai3 in a small cohort of adenocarcinoma (N=60), and its role in cell proliferation and Ca^2+^ influx in lung cancer cell lines [21]. Here we evaluated the immunohistochemical expression of Orai3 in a large cohort of lung adenocarcinoma samples (N=200) taking into account their clinic-pathologic features (tobacco exposure, tumour necrosis, visceral pleural invasion, 2011 classification of lung adenocarcinoma and stage), TTF1 expression, tumoural aggressiveness (ERα expression, KRAS and EGFR mutations) and prognostic significance was further evaluated.

## RESULTS

### Orai3 is overexpressed in lung cancer and associated with tumoural aggressiveness

**Figure 1:**
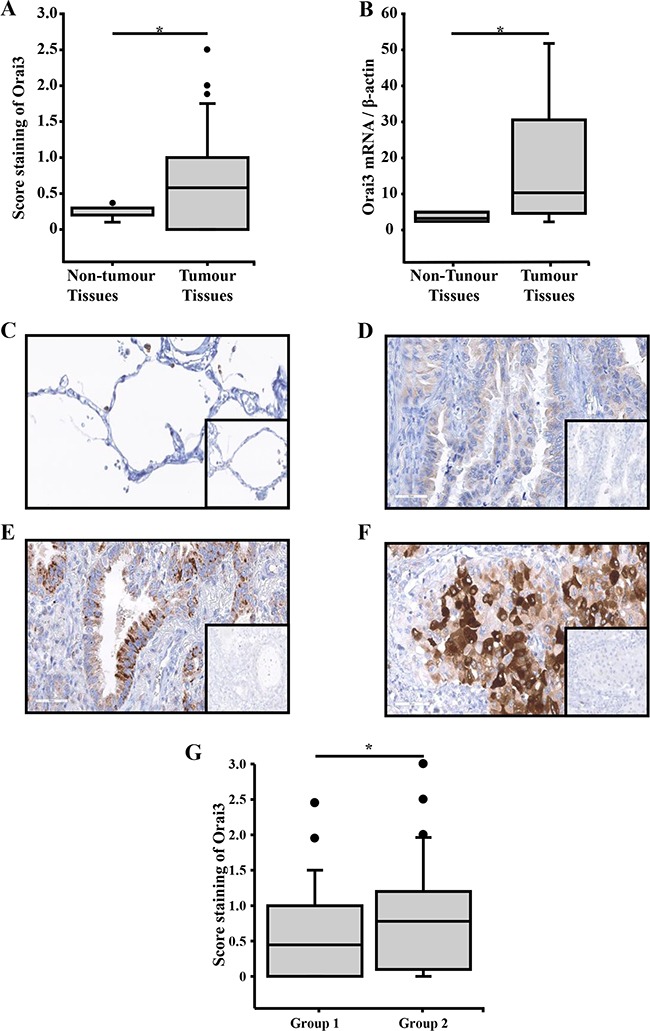
Immunohistochemical stainings of Orai3 in lung adenocarcinoma tissues according to the lung adenocarcinoma classification and relative quantity of Orai3 mRNA in lung adenocarcinoma tissues **A.** In 200 specimens, the score of Orai3 was higher in tumour tissues than in matched non-tumour tissues (*p<0.05,* Wilcoxon signed-rank test). **B.** Orai3 mRNA expression in 10 formalin-fixed, paraffin-embedded lung adenocarcinoma and non-tumour tissue samples (*p<0.05*, Mann-Whitney test). **C.** Normal lung tissue showed a few staining of Orai3 in all the epithelium cells (x200). **D.** Lung adenocarcinoma showed a few staining of Orai3 in invasive adenocarcinoma lepidic and papillary predominant (x200). **E.** Lung adenocarcinoma showed a moderate Orai3 staining in infiltrating adenocarcinoma acinar predominant (x200). **F.** Lung adenocarcinoma showed high Orai3 staining in infiltrating adenocarcinoma solid predominant (x200); inserts show negative controls obtained by omitting the primary antibody. **G.** The assessment of Orai3 score was performed on 180 invasive adenocarcinoma samples. The group 1 includes samples of invasive adenocarcinoma lepidic, papillary and acinar predominant. The group 2 includes samples of invasive adenocarcinoma solid and micropapillary predominant.

First, we confirmed that Orai3 is overexpressed in lung adenocarcinomas (Figure 1A). Among the 200 cases of tumour tissues matched with non-tumour tissues tested, the score of Orai3 staining in tumour tissues was higher than in matched non-tumour ones (non-tumour tissues: 0.3 ± 0.04 *vs.* tumour tissues: 0.66 ± 0.005, *p<0.05*; Wilcoxon signed-rank test). We found also that the mRNA for Orai3 was strongly overexpressed in tumour tissues (4.08 ± 0.54 folds higher than in non-tumour tissues, Figure 1B, *p<0.05*; Mann-Whitney). The expression of Orai1 and Orai2 was also analyzed by immunohistochemistry in the same tissues samples (N=200). In contrast to the over-expression of Orai3 in 66.5% (N=133/200, H-score mean=0.66), Orai1 and Orai2 overexpression were found in 31% (N=62/200, H-score mean=0.20) and 32% (N=64/200, H-score mean=0.19) cases of the tissue samples studied respectively. Representative expressions of Orai1 and Orai2 in cancerous human lung tissues are showed in the Supplementary Figure S1.

We further investigate whether the expression of Orai3 is associated to tumour aggressiveness. To do this, we compared the score staining of Orai3 in invasive lepidic, papillary, acinar and solid adenocarcinomas. The staining of Orai3 was low in non-tumour lung, and in invasive predominant lepidic and papillary adenocarcinoma (Figure 1C–1D), was moderate in infiltrating acinar adenocarcinoma (Figure 1E), and high in infiltrating solid adenocarcinoma (Figure 1F). We quantified the score staining of Orai3 in two groups: group 1 (invasive adenocarcinoma lepidic, papillary and acinar predominant) and group 2 (invasive adenocarcinoma solid and micropapillary predominant). Score staining of Orai3 was higher in group 2 than in group 1 (Figure 1G: 0.64 ± 0.007 for the group 1, N=133/180 *vs*. 0.95 ± 0.02 for the group 2, N=47/180; *p<0.001).* Thus, a high expression of Orai3 was significantly associated with solid and micropapillary predominant invasive adenocarcinomas which are the more aggressive of lung adenocarcinoma. These data suggest that Orai3 expression is associated with tumour aggressiveness.

### Clinical significance of Orai3 expression in lung cancer patients

The relationship of Orai3 expression and clinico-pathological factors from 200 lung cancer patients is presented in Table 1. Univariate and multivariate regression analysis revealed that high expression of Orai3 was significantly associated with tumour necrosis, tobacco use, TTF1 expression, and visceral pleural invasion (OR: 5.03) and ERα expression (OR: 5.29) respectively (Table 1). Moreover, of the 59 samples exhibiting high expression of Orai3, a high ERα was found in all samples (100%). A representative immunohistochemistry staining is shown in Supplementary Figure S2.

**Table 1:** Association between Orai3 expression and clinico-pathological characteristics of the lung adenocarcinoma (N=200)

Variables	Univariate analysis		Multivariate analysis	
	OR + 95% CI	*p* value*	OR + 95% CI	*p* value*
Necrosis (p *vs.* a)	2.18 [1.12-4.28]	0.023	1.15 [0.60-2.20]	0.66
Tobacco use (s *vs.* nos)	1.67 [0.99-2.81]	0.04	1.89 [0.473-8.03]	0.39
VPI (p *vs.* a)	5.16 [2.30-11.55]	<0.001	4.68 [1.9-11.51]	<0.001
Histologic classification† (M-S *vs.* L-P-A)	3.15 [1.42-6.98]	0.005	1.40 [0.47-4.11]	0.55
TTF1 (> 1% *vs.* < 1%)	2.08 [1.01-4.26]	0.04	2.33 [0.91-5.97]	0.08
ERα (> 1% *vs.* < 1%)	7.72 [3.42-17.39]	<0.001	5.29 [2.05-13.67]	<0.001
EGFR (MT *vs.* WT)	0.72 [0.178-2.99]	0.66		
KRAS (MT *vs.* WT)	3.03 [0.36-25.21]	0.30		
Stage (IIIa-IV *vs.* I-II)	1.89 [0.89-4.01]	0.097		

* Univariate and multivariate logistic regression model † Novel classification of lung adenocarcinoma from WHO 2015 Abbreviation: a: absence; ERα: estrogen receptor alpha; MT: mutant type; L-P-S-M-S: invasive adenocarcinoma predominant lepidic, papillary, acinar, micropapillary and solid; nos: non-smokers; OR: Odd Ratio; p: presence; s: smokers; TTF1: thyroid transcription factor 1; VPI: visceral pleural invasion; WT: wild type.

**Figure 2:**
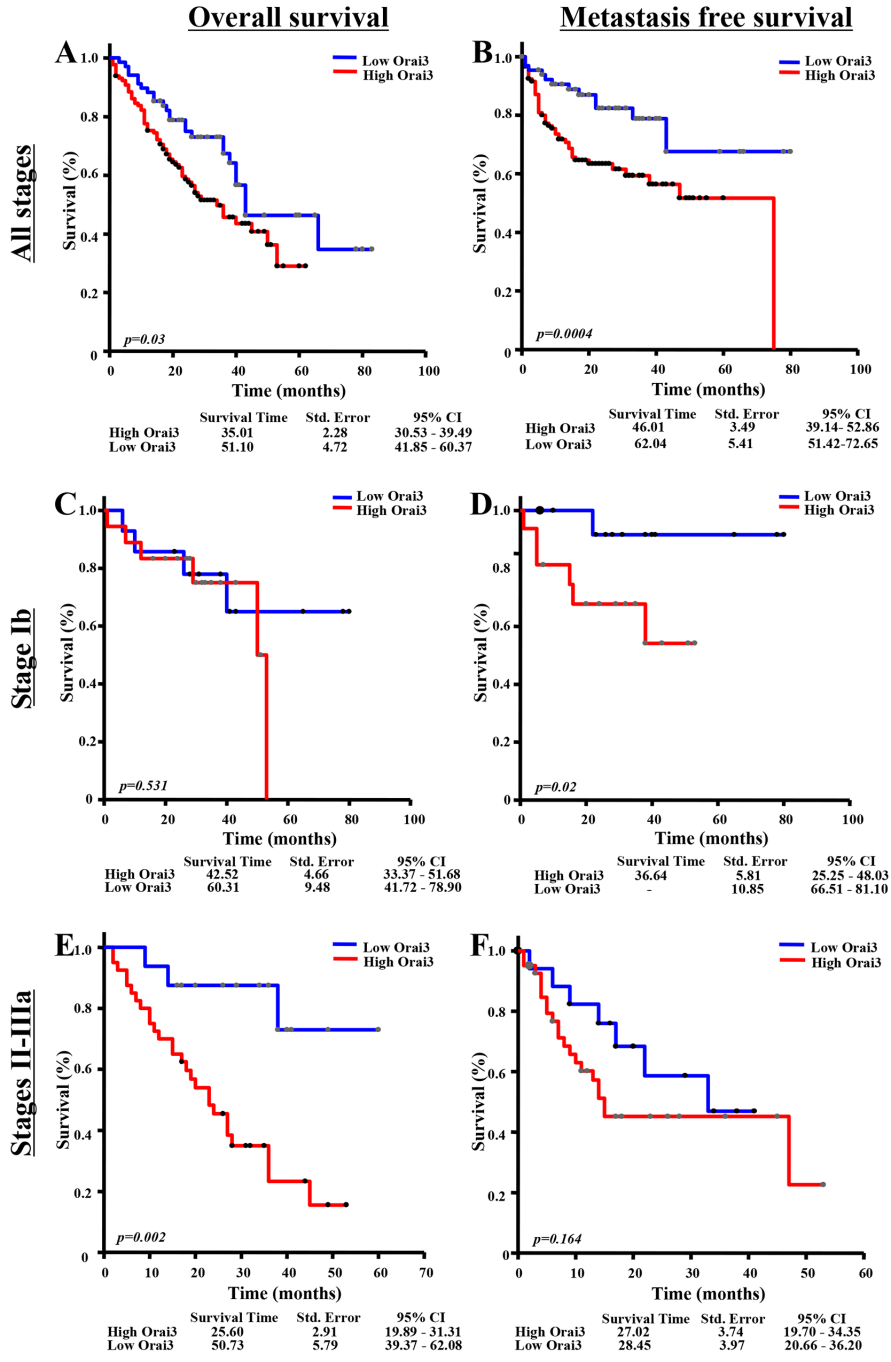
Association of Orai3 expression with survival (A-B) All stages (N=200): **A.** Overall survival of patients with high Orai3 expression compared with low Orai3 expression (*p<0.05*, Log rank test). **B.** Metastasis free survival of patients with high Orai3 expression compared with low Orai3 expression (*p<0.05,* Log rank test). **(C-D)** With stage Ib disease (N=32): **C.** Overall survival of patients with high Orai3 expression compared with low Orai3 expression (ns; Log rank test). **D.** Metastasis free survival of patients with high Orai3 expression compared with low Orai3 expression (*p<0.05*, Log rank test). **(E-F)** With stage II-IIIa disease (N=62): **E.** Overall survival of patients with high Orai3 expression compared with low Orai3 expression (*p<0.05*, Log rank test). **F.** Metastasis free survival of patients with high Orai3 expression compared with low Orai3 expression (ns, Log rank test). Low Orai3 expression group is shown as blue solid line; high Orai3 expression group is shown as red solid line. Abbreviations: 95% CI: 95% confidence interval; ns: non-significant; Std. Error: standard error.

However, no association between score staining of Orai3 and EGFR mutation (9/129), KRAS mutation (18/52) and stage TNM was found (Table 1). The overall survival (OS) and metastasis free survival (MFS) rate in the high Orai3 expression group was significantly lower in comparison with the low Orai3 expression group (Figure 2A). For OS, the median follow-up was 35.01 months *vs*. 51.11 months for the high and low Orai3 expressions respectively (Figure 2A, *p<0.05;* log rank test). For the metastasis free survival, the median was 46.01 months *vs*. 62.04 months for the high and low Orai3 expressions respectively (Figure 2B, *p<0.05*, log rank test). Multivariate regression analysis revealed that high expression of Orai3 is an independent prognostic factor for lung cancer outcome, as the tumour stage (Table 2).

**Table 2:** Cox regression analysis for OS and MFS for adenocarcinoma patients for the whole cohort (N=200)

Variable	OS				MPF			
	Univariate analysis		Multivariate Analysis		Univariate analysis		Multivariate analysis	
	HR [95%CI]	*p* value	HR [95%CI]	*p* value	HR [95%CI]	*p* value	HR [95%CI]	*p* value
Orai3 (high *vs.* low)	1.66 [1.04-2.63]	0.033	1.55 [1.02-2.23]	0.045	2.82 [1.54-5.17]	0.001	2.73 [1.45-5.16]	0.002
Gender (M *vs.* F)	0.85 [0.45-1.60]	0.570			0.82 [0.44-1.54]	0.516		
Stage (I-IV *vs.* Ia)		<0.001		<0.001		<0.001		<0.001
Ib *vs.* Ia	0.67 [0.33-1.37]		0.82 [0.33-2.04]		1.29 [0.57-2.92]		1.94 [0.85-4.45]	
IIa *vs.* Ia	0.87 [0.36-2.07]		0.86 [0.29-2.54]		2.85 [1.23-6.64]		3.25 [1.36-7.77]	
IIb *vs.* Ia	1.73 [0.80-3.76]		1.79 [0.66-4.80]		3.08 [1.28-7.45]		3.57 [1.44-8.81]	
IIIa *vs.* Ia	2.84 [1.62-4.96]		3.01 [1.58-5.73]		3.89 [1.95-7.77]		2.51 [1.18-5.34]	
IV *vs.* Ia	4.03 [2.09-7.79]		4.73 [2.32-9.63]		10.94[5.23-2.90]		10.86 [5.01-3.50]	
Necrosis (p vs. a)	1.48 [0.91-2.42]	0.116	1.06 [0.78-1.44]	0.609	1.72 [1.06-2.80]	0.029	1.53 [0.92-2.54]	0.099
Age	0.99 [0.97-1.02]	0.884			0.99 [0.97-1.02]	0.369		
Tobacco use (s *vs.* nos)	1.86 [0.58-5.96]	0.740			0.94 [0.36-2.47]	0.590		

Abbreviations: a: absence; 95%CI: 95% confidence interval; F: female; HR: Hazard Ratio; M: male; MFS: metastasis free survival; ns: non-significant; nos: non-smokers; OS: overall survival; p: presence; s: smokers.

### Expression of Orai3 and survival in stages Ib and II-III

The chemotherapy is recommended for patients with stage II or III of lung adenocarcinoma, and also for patients with stage Ib with high risk of recurrence. As Orai3 confers survival of cancer cells [21], we evaluated the expression of Orai3 in stage Ib and II-III.

For Ib stage, there was no significant impact of Orai3 expression on OS (Figure 2C). For MFS, the median of high Orai3 expression group was lower than low Orai3 expression group (Figure 2D: 36.64 months *vs*. >80 months, *p<0.05;* log rank test). Univariate Cox proportional hazards analysis also showed a negative impact of Orai3 expression on MFS for patients with stage Ib (Table 3: HR: 8.01, *p=0.049*), while no significant difference for OS was found (Table 3). For II-IIIa tumour stages, a negative impact of Orai3 expression was observed on OS (Figure 2E, median 25.60 months for high Orai3 *vs*. 50.73 months for low Orai3 expression, *p=0.02*; log rank test), but not on MFS (Figure 2F). The multivariate Cox proportional hazards model confirms the negative impact of high Orai3 expression for the overall survival of patients with stages II-IIIa (Table 4: HR: 7.16, *p=0.008*). No significant difference was observed for MFS.

**Table 3:** Cox regression analysis for OS and MFS for adenocarcinoma patients for patients with Ib stage (N=32)

Variable	OS				MPF			
	Univariate Analysis		Multivariate analysis		Univariate analysis		Multivariate analysis	
	HR [95%IC]	*p* value	HR [95%IC]	*p* value	HR [95%IC]	*p* value	HR [95%IC]	*p* value
Orai3 (high *vs.* low)	0.32 [0.03-3.10]	0.533			8.01 [1.02-64.19]	0.049		
Gender (M *vs.* F)	1.88 [0.17-20.37]	0.944			2.26 [0.24-21.48]	0.581		
Necrosis (p *vs.* a)	3.71 [0.62-22.28]	0.152			3.19 [0.48-21.13]	0.265		
Age	0.94 [0.79-1.09]	0.929			1.08 [1.01-1.16]	0.242		
Tobacco use (s *vs.* nos)	4.35 [0.00-28.51]	0.652			0.32 [0.00-35.89]	0.999		

Abbreviations: a: absence; 95%IC: 95% confidence interval; F: female; HR: Hazard Ratio; M: male; MFS: metastasis free survival; nos: non-smokers; OS: overall survival; p: presence; s: smokers.

**Table 4:** Cox regression analysis for OS and MFS for adenocarcinoma patients for patients with II-IIIa stage (N=62)

Variable	OS				MPF			
	Univariate analysis		Multivariate Analysis		Univariate analysis		Multivariate analysis	
	HR [95%IC]	*p* value	HR [95%IC]	*p* value	HR [95%IC]	*p* value	HR [95%IC]	*p* value
Orai3 (high *vs.* low)	4.61 [1.61-13.17]	0.004	7.16 [1.69-30.34]	0.008	1.82 [0.78-4.25]	0.168	1.37 [0.57-3.28]	0.479
Gender (M *vs.* F)	1.16 [0.42-3.19]	0.334			0.66 [0.27-1.61]	0.307		
Necrosis (p *vs.* a)	1.98 [0.91-4.34]	0.086	1.72 [0.78-3.78]	0.176	3.20 [1.42-7.20]	0.005	2.94 [1.29-6.71]	0.011
Age	1.02 [0.97-1.06]	0.379			0.96 [0.92-1.01]	0.221		
Tobacco use (s *vs.* nos)	1.29 [0.26-6.55]	1			0.33 [0.09-1.17]	0.824		

Abbreviations: a: absence; 95%IC: 95% confidence interval; F: female; HR: Hazard Ratio; M: male; MFS: metastasis free survival; nos: non-smokers; OS: overall survival; p: presence; s: smokers.

## DISCUSSION

Here, we confirm the association of the Orai3 expression with invasive adenocarcinoma, micropapillary and solid predominant subtypes, in a large cohort of patients of lung cancer. Moreover, we report, for the first time, that high expression of Orai3 is an independent prognostic factor for lung adenocarcinomas. The expression of Orai3 has been evaluated using immunohistochemistry, which is the more relevant technique for heterogeneous tumour [22]. Indeed, in previous studies lung adenocarcinoma exhibited higher heterogeneity [23], and intra-tumor variation of expression in lung cancer was reported by immunohistochemistry [24]. The excellent specificity of Orai3 antibody used in this work has been previously reported [21]. Moreover, the overexpression of Orai3 in lung tissue was also confirmed by qPCR on the same samples embedded by paraffin.

The role of Orai3 in cell proliferation and calcium homeostasis in lung cancer cell lines was previously reported by our group [21]. Indeed, silencing of Orai3 (using siRNA against Orai3) accumulates cells in G0/G1 phase of the cell cycle, reduces calcium entry leading to cell cycle arrest and cell proliferation inhibition [21]. Studies on the association between the expression of ion channels and the prognosis of lung adenocarcinoma patients emerge. Recently, a transcriptomic analysis has compared the expression of ion channel encoding genes of normal and lung adenocarcinoma tissues. 37 ion channels genes were identified as being differentially expressed between the two groups [25]. However, the Orai isoforms were not assessed in this study. The authors have established a risk score based on the expression of the differentially expressed genes in order to investigate the prognostic of such ion channels genes. Using multivariate analysis, they found that the risk score is an independent prognostic factor for survival [25]. Moreover, they found the risk score was higher for ever-smokers than for never-smokers [25]. The same group has reported that VDAC1 (voltage-dependent anion channel type 1) is associated with shorter overall survival and turned out to be an independent prognostic factor of recurrence in multivariate analysis [26]. Our results show that high Orai3 expression was associated with poor prognosis (OS: 35.01 months *vs*. 51.11 months and MFS: 46.01 months *vs*. 62.04 months).

Furthermore, we showed that Orai3 expression is also associated with ERα expression in lung adenocarcinoma such as demonstrated in breast adenocarcinoma [19]. Indeed, in breast cancer, Orai3 is overexpressed and its expression is under the control of estrogen receptor alpha (ERα) that regulates Ca^2^^+^ entry, cell proliferation and survival of estrogen receptor-positive MCF-7 cell line [16, 17]. ERα is expressed in both tumor tissue and cultured non-small cell lung cancer (NSCLC) cell lines. 17-β-estradiol increased cell proliferation *in vitro* and *in vivo* [27]. Moreover, suppressing expression of ERα elicits a significant reduction in NSCLC cell proliferation *in vitro* [30, 31]. Furthermore, the association of ERα with lung cancer is well established [28]. Clinically, the over-expression of ERα is associated with a poor prognosis in lung adenocarcinoma patients both for men and women [10, 29-31]. In animal model, enhanced E2/ERα facilitates the smoking carcinogen N-nitrosamines-mediated lung carcinogenesis [32]. We demonstrated here an association between Orai3 expression and tobacco exposure. These findings suggested that Orai3 could be associated with tobacco smoke mediated ERα expression.

Postoperative chemotherapy is given to slow down or stop the growth of cancer cells. Even after a cancer has been removed with surgery, cancer cells can remain in the body, increasing the risk of relapse. Chemotherapy can eliminate these cancer cells and increase the chance of cure, but it is prescribed only to patients with a risk of recurrence high enough to counterbalance the side effects of chemotherapy. It is recommended for patients with stages II or III NSCLC and for some (but not all) patients with stage I disease. Reasonably the use of chemotherapy after radical surgery is justified by increasing overall survival from 60 to 64% [5-7]. As the benefit of chemotherapy is moderate, it is important to select appropriate patients with resectable tumours. Thus, patients are selected by using biomarkers that predict prognosis [33]. Adjuvant chemotherapy is prescribed only to the selected patients who are likely to benefit from the treatment [34, 35]. The outcomes have showed that high Orai3 expression was associated with poor prognostic for MFS in Ib stage and with poor prognosis for OS in II-III stage. This result supports high Orai3 expression as a good biomarker to select appropriate patients with Ib-II-III stage. Furthermore, the fact that higher Orai3 expression was found in III-IV stages *vs*. I-II stages (N=154, 0.69±0.06 *vs.* N=46, 0.81±0.12) support the use of Orai3 as therapeutic target.

Growing studies show the Orai3 pivotal role in mediating tumorigenesis. Indeed, two preclinical studies, in murine models, in breast and prostate cancers respectively have highlighted that inhibition of Orai3 impairs tumor growth and metastasis [36, 37] suggesting Orai3 as a useful therapeutic target in oncology. Recently, a new compound targeting calcium release-activated calcium (CRAC) channels including Orai1 and Orai3 channels has been developed by Rhizen pharmaceuticals for the treatment of the non-small cell lung cancer (Patent US 2011/0112058 A1, [38]). The compound named “B” decreases the expression of both Orai1 and Orai3 in NCl-H460 lung cancer cell line, and tumour growth *in vivo* [38, 39]. Although, this compound has affected both Orai1 and Orai3 expressions, these outcomes suggest that this drug could be a promising approach for cancer lung adenocarcinoma treatment.

In conclusion, Orai3 is associated with poor prognosis of lung adenocarcinoma and might be used as a novel prognostic marker for a chemotherapy treatment indication in early stage adenocarcinoma.

## MATERIALS AND METHODS

### Ethics statement

This study was conducted according to “Comité Consultatif de Protection des Personnes dans la Recherche Biomédicale de Picardie” in which 200 patients in resectable stage of lung adenocarcinoma were identified from the database of pathology department of Amiens University Hospital. Surviving patients provided their written consent for their clinical information to be included in the study.

### Specimens of study

Patients with tumour and non-tumour samples between 2008 and 2013 have been included. Data entry was finalized on 2015. The tumors were classified according to the 2011 WHO classification [8]. 200 patients with lung adenocarcinoma were included at the time of the last follow-up. The median age of the patients at initial diagnosis was 60.5 years (range: 34-84 years). The majority were male (73%, N=143/200). 86.9% (N=173/200) of patients had tobacco exposure, defined by more than 100 cigarettes smoked in their lifetime.

### Immunohistochemical analysis

The method for detecting and determining the expression of different proteins (Orai3 and estrogen receptor alpha) has been previously described [21]. Briefly, formalin-fixed, paraffin-embedded 4 μm sections of the lung tissue were first deparaffinised in xylene and rehydrated in ethanol. The endogenous peroxidase activity was blocked (Ventana) before the antigen retrieval. The Cell Conditioning Solution CC1 (Ventana, BenchMark) was then used for antigen retrieval.

Immunohistochemical staining was carried out on BenchMark ULTRA (Ventana), using antibody directed against Orai3 (rabbit polyclonal, 1:200 dilution, Sigma, Saint Louis, USA) and antibody directed against Estrogen Receptor Alpha (ERα) (SP-1, Ventana, prediluted, Meylan, France). This was followed by the avidin-biotin-peroxidase complex technique. Reactions were developed using a chromogenic reaction in DAB (diamino-3,3benzidinetetrahy-drochloride) substrate solution (iVIEW DAB Detection Kit, Ventana). The tissues were counterstained with hematoxylin. The antibody is certified for immunohistochemistry by Sigma Inc. A negative control was performed using the same technique without the primary antibody.

### Quantification of staining expression by immunohistochemistry

Independent observers quantitatively evaluated staining expression (x40 objective and x10 eyepiece) of each specimen. To evaluate relative immune-intensity in lung adenocarcinoma, immunoreactivity was also evaluated by H scoring system [40]. Briefly, staining carcinoma cells were further classified into the strongly or weakly positive cells, and H scores were subsequently generated by adding together 3x % strongly stained cells, 2x % moderate stained cells 1x % weakly stained cells, and 0x % negative cells. Thereafter, this staining score of Orai3 expression is referred to as the Orai3 expression level. Cases with ERα of > 1% were considered ERα-positive for lung adenocarcinoma.

### Quantitative RT-PCR

Every DNA sample was obtained from two 5 μm-thick sections of non-tumour and tumour tissues. Extractions were performed manually according to manufacturers' protocols (ReliaPrep FFPE Total RNA Miniprep System, Promega Corp, France). Total RNA (1 μg) was reverse transcribed into cDNA with random hexamers and MutliScribesTM Reverse Transcriptase (Applied Biosystems, Carlsbad, CA) as previously described [41]. Quantitative RT-PCR was performed on a Light Cycler system (Roche, Basel, Switzerland) using a mix containing SYBR green (Applied Biosystem, Carlsbad, CA). For the PCR reaction, sense and anti-sense PCR primers specific to Orai3 (for 5’-AAGTCAAAGCTTCCAGCCGC-3’; and rev 5’-GGTGGGTACTCGTGGTCACTCT-3’) and β-actin (for 5’-CAGAGCAAGAGAGGCATCCCT-3’; and rev5’-ACGTACATGGCTGGGGTG-3’) were used. The relative amount of Orai3 “target” was normalized to the endogenous control (β-actin) and compared to the reference sample (non-tumour) using the Pfaffl method.

### Statistical analysis

Data are presented as Mean±SEM. The Mann-Whitney U test was used to compare the Orai3 score between patients according to the presence or absence of variable, and the Wilcoxon signed-rank test was used to compare the distribution of expression among tissue types (non-tumour and tumour). Median survival was computed from the date of diagnosis to the date of the patient's last known vital status. The time to first metastasis was computed from the date of stage diagnosis to the date of first metastasis. A univariate and multivariate logistic regression model was built that included the following factors: tumour necrosis (presence *vs*. absence), tobacco exposition (smoker *vs*. no smoker), pleural visceral invasion (presence *vs*. absence), histologic classification of invasive adenocarcinoma (micropapillary or solid *vs*. lepidic, acinar or papillary predominant), expression of ERα (expression > 1% *vs*. < 1%), expression of TTF1 (expression > 1% *vs*. < 1%), EGFR (MT (Mutant Type) *vs*. WT (Wild Type)) and KRAS (MT *vs*. WT). This model was used to predict Orai3 expression. The Orai3 was considered first as a continuous variable, and secondary analyses were performed separately which categorized patients as having p-STAT3 expression < 1% *vs.*> 1% [17]. The log-rank test was used to assess groups with low Orai3 expression (< 1%) *vs.* high *(*> 1%). Cox proportional hazards regression was used to analyze the association between Orai3 expression and survival or the development of metastasis. We decided on this dichotomization based on its possible clinical relevance after analyzing absolutely none versus any Orai3 expression as well as comparing percentiles that represented the highest and lowest levels of expression. A p value of less than 0.05 was considered significant. All statistical analysis was performed using R.

## SUPPLEMENTARY MATERIALS FIGURES


