# Supplementary material for: Orai3 is a predictive marker of metastasis and survival in resectable lung adenocarcinoma
Source: Oncotarget. 2016 Nov 7;7(49):81588–97. doi: 10.18632/oncotarget.13149 (PMC5348414; doi:10.18632/oncotarget.13149)
Supplement: Supplementary file 1 [file oncotarget-07-81588-s001.pdf]

Orai3 is a predictive marker of metastasis and survival in resectable lung adenocarcinoma

SUPPLEMENTARY FIGURES

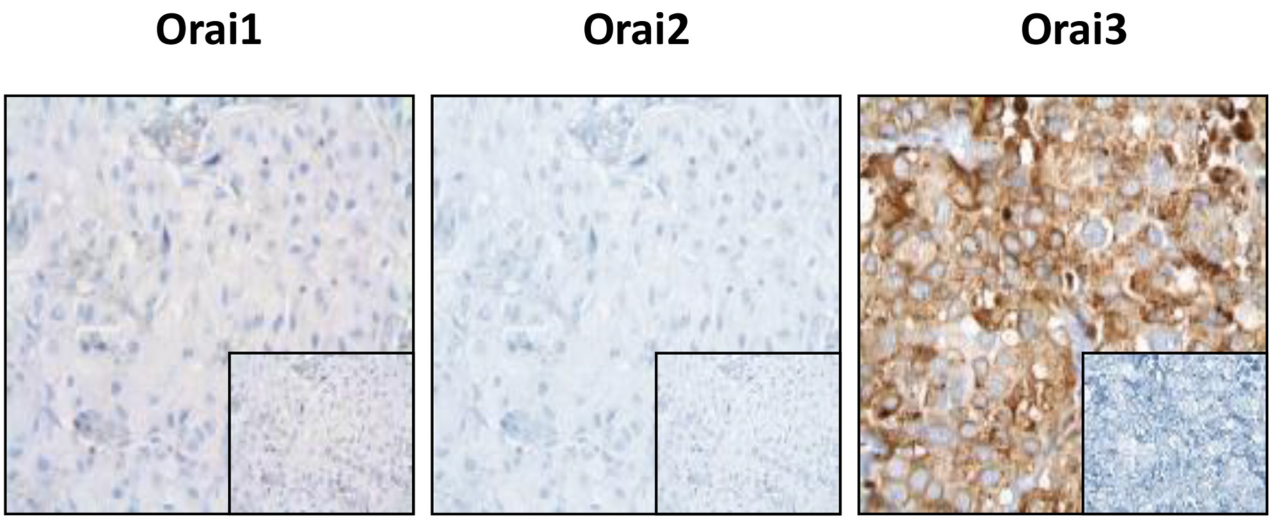

Supplementary Figure S1: Orai3 was the most expressed Orai isoforms in lung adenocarcinoma. Representative examples of Orai1, Orai2 and Orai3 staining in cancerous lung tissues, as assessed by immunohistochemistry. *Insert* negative controls obtained by omitting the primary antibody. We observed a weak staining of both Orai1 and Orai2 and a strong staining of Orai3 among 200 lung adenocarcinoma samples.

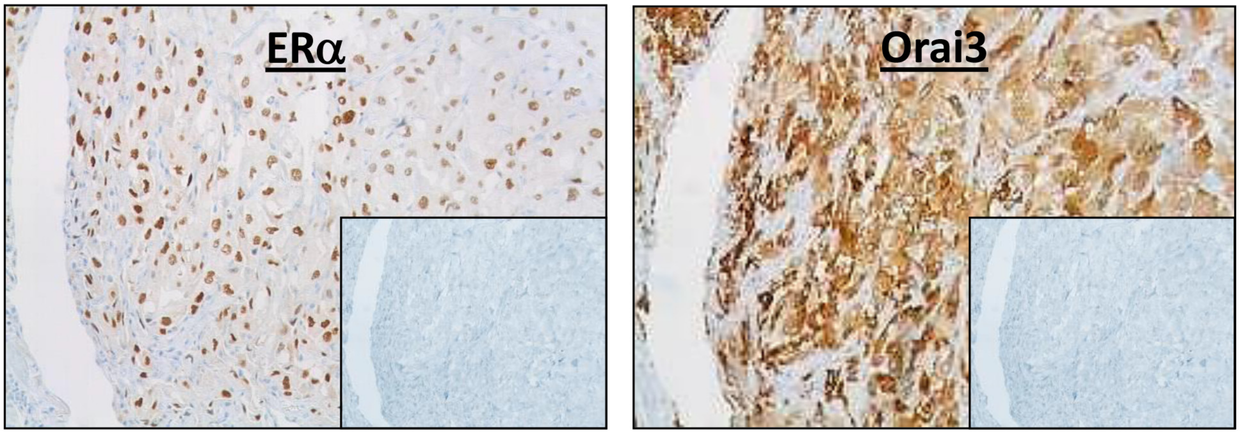

Supplementary Figure S2: Orai3 and ERα are co-expressed in lung adenocarcinoma. Typical immunohistological staining of ERα and Orai3 in lung cancer tissues from the same patient. *Insert:* the same field without staining when the primary antibody was omitted represents the negative control.
